# Bromocresol Green/Mesoporous Silica Adsorbent for Ammonia Gas Sensing via an Optical Sensing Instrument

**DOI:** 10.3390/s110404060

**Published:** 2011-04-06

**Authors:** Yu-Chang Chang, Hsunling Bai, Shou-Nan Li, Chun-Nan Kuo

**Affiliations:** 1 Institute of Environmental Engineering, National Chiao Tung University, Hsinchu 30010, Taiwan; E-Mail: bruce0313@gmail.com; 2 Green Energy and Environment Research Laboratories, Industrial Technology Research Institute, Hsinchu 31040, Taiwan; E-Mails: SNLi@itri.org.tw (S.-N.L.); chunnankuo@itri.org.tw (C.-N.K.)

**Keywords:** ammonia gas, optical sensor, mesoporous silica material, UV-Vis absorbance, bromocresol green dye

## Abstract

A meso-structured Al-MCM-41 material was impregnated with bromocresol green (BG) dye and then incorporated into a UV-Vis DRA spectroscopic instrument for the online detection of ammonia gas. The absorption response of the Al-MCM-41/BG ammonia sensing material was very sensitive at the optical absorption wavelength of 630 nm. A high linear correlation was achieved for ppmv and sub-ppmv levels of ammonia gas. The response time for the quantitative detection of ammonia gas concentrations ranging from 0.25 to 2.0 ppmv was only a few minutes. The lower detection limit achieved was 0.185 ppmv. The color change process was fully reversible during tens of cycling tests. These features together make this mesoporous Al-MCM-41 material very promising for optical sensing applications.

## 1. Introduction

Sensing low concentrations of chemical vapors is an area of great interest with many practical applications. In semiconductor and optoelectronic manufacturing, a small amount of ammonia gas can lead to serious damage to electronic devices and decrease product yields [1]. Thus, in these industries ammonia gas detection at sub-ppmv levels is required for ensuring product quality. For environmental purposes, ammonia can be smelled at a level higher than 0.04∼57 ppmv [2]. It irritates eyes, throats and noses and affects human health when it reaches the 50–100 ppmv level [3]. In order to avoid hazards, the US Occupational Safety and Health Administration (OSHA) limits worker ammonia exposure to 50 ppmv over a 8 h work day and 40 h per week [4], while the US National Institute of Occupational Safety and Health (NIOSH) proposes that the ammonia concentration should not exceed 25 ppmv over 10 h per day or 40 h per week in the workplace [5].

Several different techniques have been developed for sensing ammonia gas. Conventional real-time gas monitoring has commonly been performed employing electrochemical sensors [6]. However, such techniques have inherent disadvantages such as the need for reference electrodes and the development of surface potentials, *etc.* Moreover, they can’t monitor low ammonia concentrations because of the high bias. Many of these problems can be solved by employing optical sensors using chemical sensing materials. Optical sensors have attracted much attention because of their accuracy and high sensitivity to low ammonia gas concentrations. Besides, the detection can be achieved without any direct contact between the expensive optical parts and the corrosive ammonia gas samples [7]. Such features may lead to wide acceptance in real applications, and provide a solution for detecting ammonia at sub-ppmv levels.

Many different materials have been employed to entrap the dyes used for ammonia detection. For instance, Markovics *et al.* [8] used bromocresol green indicators immobilized on an anodized aluminum substrate, and found that they could measure 10–90 ppmv ammonia gas in a few seconds. Although this material had the advantage of a fast response time, its lower detection limit was still too high for practical use. Courbat *et al.* [9] proposed another material, bromophenol blue-doped poly(methyl methacrylate), which could be used to detect ammonia in sub-ppmv levels. However, the quantitation time in their study was over 30 min. An ideal sensing material for optical sensors should offer some advantages such as a fast response (*i.e*., less than 10 min), high sensitivity, and be inexpensive in order to achieve widespread routine use. Thus, new materials are required to meet these needs.

More recently, ordered mesoporous silica materials have become of interest as chemical sensing materials due to their uniform porosity, mechanical stiffness, thermal stability, high surface areas and pore volumes, tunable pore sizes and the possibility of incorporation of hetero-atoms into the silica structure [10–12]. Fiorilli *et al.* [13] and Onida *et al.* [14] used Reichardt’s dye impregnated on mesoporous SBA-15 to develop ammonia sensing materials. Tao *et al.* [15] used an organic dye-impregnated porous silica material to detect 1–5 ppmv ammonia gas in 20 min. Nevertheless, to the authors’ knowledge, no literature has discussed employing ordered mesoporous silica materials as chemical sensing materials for the quantification of ammonia gas at sub-ppmv levels.

The purpose of this study was to develop a mesoporous silica material which could measure ammonia gas concentrations at sub-ppmv levels. In this work, a highly sensitive and fully reversible ammonia sensing material is proposed using bromocresol green, an organic dye, impregnated on mesoporous Al-MCM-41(50). Since the color of the dye-impregnated Al-MCM-41(50) will change during ammonia adsorption, it can be incorporated into a UV-Vis Diffuse Reflection Accessory (DRA) spectroscopic instrument for the online detection of ammonia gas. The color change process was repeated for tens of cycles to check for the reversibility of the sensing material on the ammonia gas detection.

## 2. Experimental

### 2.1. Preparation of Mesoporous Al-MCM-41

Mesoporous Al-MCM-41(nSi/nAl = 50) molecular sieves were synthesized by a hydrothermal treatment method based on our prior experience [16]. Cetyltrimethylammonium bromide (CTAB, C_19_H_42_BrN) was employed as the structure-directing template in the synthesis. The molar composition of the gel mixture was 1 Na_2_SiO_3_:0.005 Al_2_O_3_: 0.11 C_19_H_42_BrN:0.35 H_2_SO_4_: 40.3 H_2_O. In a typical synthesis procedure, 21.2 g of sodium metasilicate (Kanto Chemical Co. Inc., Japan) was dissolved in 80 mL de-ionized (DI) water and then combined with aluminum sulfate (0.342 g dissolved in 20 mL of DI water). The resulting mixture was stirred vigorously for 30 min. Then, approximately 11.1 mL H_2_SO_4_ (Panreac Chemicals Co., Spain) in 100 mL of DI water was added to the above mixture with constant stirring to bring down the pH to 10.5 and form a gel. After stirring, 7.2 g of CTAB (dissolved in 25 mL of DI water) was added slowly into the above mixture and the combined mixture was stirred for three additional hours. The resulting gel mixture was transferred into a Teflon coated autoclave and kept in an oven at 145 °C for 36 h. After cooling to the room temperature, the resultant solid was recovered by filtration, washed with DI water and dried in an oven at 100 °C for 6 h. Finally, the organic template was removed by using a muffle furnace in air at 550 °C for 10 h.

### 2.2. Impregnation of Bromocresol Green Indicator

The dye bromocresol green (BG, 2,6-dibromo-4-[7-(3,5-dibromo-4-hydroxy-2-methylphenyl)-9,9-dioxo-8-oxa-9λ6-thiabicyclo[4.3.0]nona-1,3,5-trien-7-yl]-3-methylphenol) is a weak organic acid whose absorbance spectrum is quite different from that of its conjugate base. The color of BG solution changes from yellow to blue over the pH range of 3.8–5.4 [17]. The Al-MCM-41(50)/BG impregnation process was evaluated under different ratios of BG to Al-MCM-41(50) as well as different process times and temperatures. The optimal dye impregnating procedure is as follows: 0.001 g of BG powder (Acros Organics Co., USA) and 100 mL of acetone (Merck & Co. Inc., Germany) were mixed and stirred for 10 min. Then 0.1 g of Al-MCM-41(50) powder was added into the mixture solution and stirred for 3 h. The resulting yellow solution was transferred into an evaporating dish and kept in an oven at 110 °C for 3–6 h. Finally, the orange colored sample of BG dye impregnated-Al-MCM-41(50) material was collected and named as Al-MCM-41(50)/BG hereafter.

### 2.3. Material Characterization

The powder X-ray diffraction patterns of Al-MCM-41 (50) and dye impregnated samples were recorded using a Panalytical X’Pert Pro MRD powder diffractometer, where a Cu target Kα-ray (operating at 30 kV and 20 mA) was used as the X-ray source. The scanning range was 2θ = 2° − 10° with 400 data points and the scanning speed was 2 °/min. The d-spacing (d_hkl_) of material was calculated by the Bragg diffraction equation.

The Brunauer-Emmett-Teller (BET) specific surface area, specific pore volume and BJH average pore diameter of the materials were measured by N_2_ adsorption-desorption isotherms at 77 K using a surface area analyzer (Micromeritics, ASAP 2020). All the materials were degassed for 6 h at 120 °C under vacuum (10^−6^ mbar) prior to the N_2_ adsorption-desorption experiments.

Transmission electron microscopy (JEOL JEM 1210, Japan) was carried out on a JEOL JEM-2010 microscope at 120 keV. The samples (5–10 mg) were ultrasonicated in ethanol and dispersed on carbon film supported on copper grids (200 mesh).

### 2.4. Online Ammonia Gas Detection

**Figure 1.**
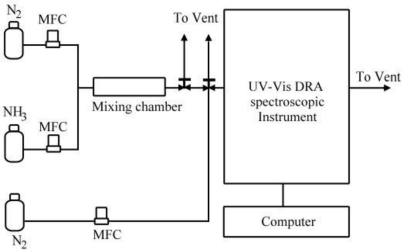
Schematic diagram of a UV-Vis DRA spectroscopic instrument for NH_3_ sensing.

The experimental set-up for the *in situ* measurements in order to test the quantitation ability of the sensing material in sub-ppmv ammonia gas is shown schematically in Figure 1. The UV-Vis spectrometer (EVOLUTION 300, Thermo Scientific, England) combined with a Diffuse Reflection Accessory (DRA, the Praying Mantis™ DRP-NI9 and HVC-VUV-4, Harrick Scientific, Pleasantville, NY, USA) monitored the optical properties of solid samples for online examination purpose and the data were recorded by a computer. The scan frequency was once per minute over the wavelength range from 400 to 850 nm.

Both NH_3_ and N_2_ gases were obtained from gas cylinders. The NH_3_ concentration was quantified by a Fourier Transform Infrared (FTIR) spectrometer (Work IR-104, ABB Bomem, QC, Canada) before each test. Then the NH_3_ and N_2_ gas streams were mixed in a mixing chamber as shown in Figure 1. All the gas flow rates were controlled by mass flow controllers (MKS, 1179A, USA), and the flow rate was re-checked by a bubble meter before each test. Different NH_3_ inlet gas concentrations were obtained by adjusting the N_2_ and NH_3_ gas flow rates to have a total gas flow rate of 400 mL/min for all test conditions. Before each test, the UV-Vis DRA spectroscopic instrument was purged with N_2_ for 20 min in order to minimize experimental errors. The temperature of this system was at room temperature (24 ± 1 °C). The relative humidity of the gas flow was monitored to be 6 ± 1% by a humidity and temperature meter (Center 310, JDC Electronic SA, Switzerland), which corresponds to an absolute humidity of less than 2,500 ppmv. The sensing material used in this experiment is in pellet form of 6 mm in diameter. And desorption process was done by heating at 150 °C for 1h to ensure total desorption.

## 3. Results and Discussion

### 3.1. Material Characterization

**Figure 2.**
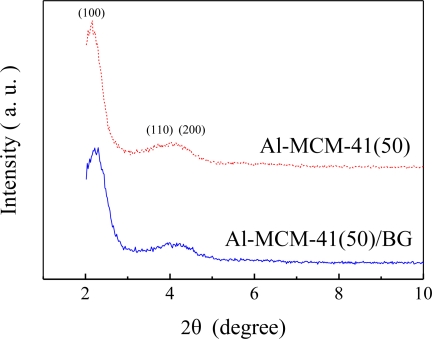
Powder X-ray diffraction pattern of Al-MCM-41(50) and dye impregnated Al-MCM-41(50)/BG.

The XRD patterns of Al-MCM-41(50) and dye impregnated Al-MCM-41(50) are shown in Figure 2. It appears that both Al-MCM-41(50) and Al-MCM-41(50)/BG present a well ordered structure and the peaks are indexed on a hexagonal lattice which corresponded to (100), (110) and (200) [10,18–20]. This indicates that the dye impregnation did not affect the ordered mesoporous structure of Al-MCM-41(50).

**Figure 3.**
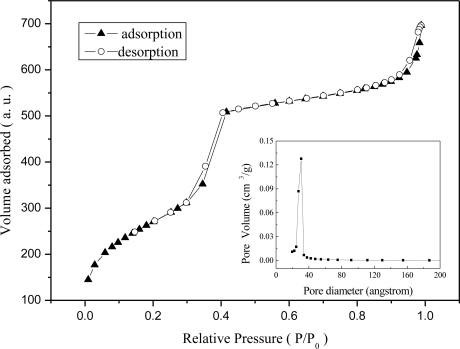
**N**itrogen adsorption-desorption isotherms of dye impregnated Al-MCM-41(50).

Figure 3 shows the N_2_ adsorption/desorption isotherms of Al-MCM-41(50) and dye impregnated Al-MCM-41(50)/BG. The isotherms are type IV according to the IUPAC classification and show an uptake of N_2_ due to capillary condensation in the relative pressure (p/p_0_) range of 0.3–0.4. Such isotherms are characteristic of mesoporous materials with a transitional pore size ranging from microporous to mesoporous. The pore size distribution of Al-MCM-41(50) was around 2.7 nm with the BET surface area of 985 m^2^/g.

**Figure 4.**
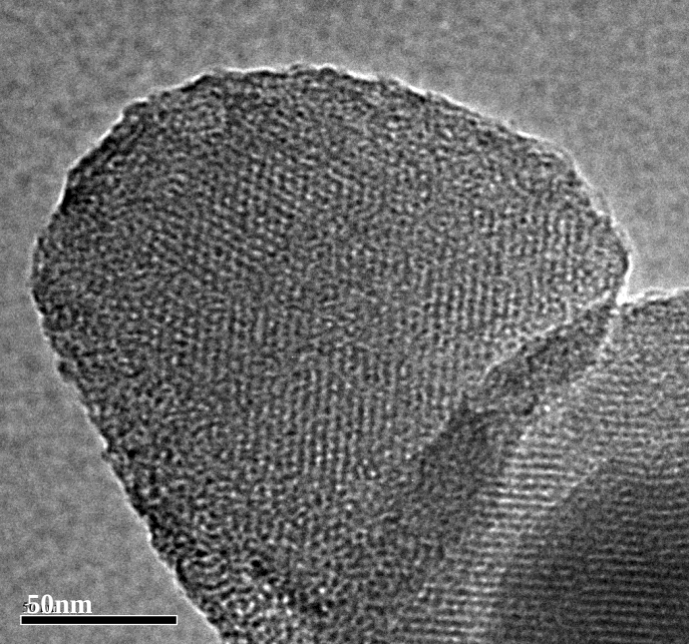
Transmission electron micrographs of dye-impregnated Al-MCM-41(50).

The TEM images of dye impregnated Al-MCM-41(50) is shown in Figure 4. The hexagonal pore structure of dye impregnated Al-MCM-41(50) was observable in the TEM images. The clear observation of the pores of dye impregnated Al-MCM-41(50) indicates that the dye molecules did not block the nano-pore structure of the host material.

### 3.2. Ammonia Gas Sensing

The sensor is based on the spectral properties of bromocresol green. Bromocresol green (tetrabromo-*m*-cresol sulfonphthalein) is an organic dye which reacts with ammonia to form an ammonium salt. Like most pH indicators, bromocresol green is a weak organic acid whose absorbance spectrum is quite different from that of its conjugate base [21]. The color of BG solution changes from yellow to blue over the pH range of 3.8–5.4, which indicates the equilibrium shifts to the de-protonated, arylmethine form of the dye [22]. This organic dye immobilized in the Al-MCM-41(50) will change its color to blue when it reacts with ammonia gas. After heating, the color of sensing material will turn back to orange.

Bromocresol green was selected based on the study of Markovics *et al.* [8], who compared three different dyes: bromophenol blue (BPB), bromocresol green (BG) and bromocresol purple (BCP) for ammonia detection. The respective pKa values are 3.8 for BPB, 4.7 for BG, and 6.0 for BCP. This study showed that the lower the pKa value of the dye, the faster the response time. However, the decrease in the pKa value of the dye also results in a longer desorption time. Thus BG should offer an appropriate compromise between the needs for fast response and fast desorption. The optical properties of the sensing materials were observed through the DRA-UV/Vis spectrometer. When a gas sample containing ammonia molecules was brought into contact with the dye-impregnated Al-MCM-41(50), ammonia molecules in the gas sample diffuse into the porous materials and react with the BG dye. This reaction reduces the concentration of BG and increases the concentration of BG salt. Thus, the absorption signal of the BG salt is increased with time upon adsorption of ammonia molecules.

**Figure 5.**
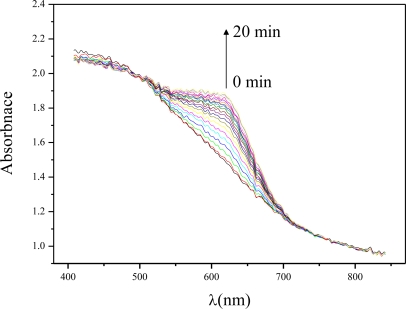
UV-Vis Spectrograms of Al-MCM-41(50)/BG as time proceeded for adsorbing NH_3_. The ammonia gas concentration was 5 ppmv.

Figure 5 shows the recorded absorption spectra change of the Al-MCM-41(50)/BG with respect to time. The absorbance increased most significantly at around 630 nm as the ammonia adsorption time increased. This peak belonged to the ammonia absorption peak. At this peak of absorption the color of the Al-MCM-41(50)/BG was blue. Therefore, the difference in the optical absorbance signal at 630 nm was monitored as the indicating signal. In the following part, the variation in the 630 nm absorbance from time zero to the test time was calculated and referred as the “absorbance difference”.

**Figure 6.**
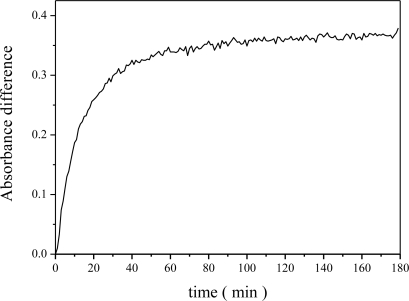
Time response on the absorbance difference of the Al-MCM-41(50)/BG for monitoring 4.3 ppmv NH_3_.

Our goal was to develop an ammonia sensing material capable of quantifying ammonia gas concentrations at sub-ppmv levels. Figure 6 shows the relationship between the absorbance difference and exposure time. The rate of change of the absorbance difference did not increase linearly. The absorbance difference increased quickly after a short NH_3_ adsorption time (0–20 min), it then increased slowly and gradually reached steady state absorbance.

When using the dye-impregnated mesoporous material for ammonia sensing, it is not recommended to operate at steady state absorbance conditions. This is because that after long NH_3_ adsorption times when steady state absorbance is reached, the sensitivity of the dye with respect to different ammonia concentrations tends to decrease, especially when it is oversaturated with ammonia. Besides, the ammonia desorption time may also increase after long ammonia adsorption time. Hence the following cyclic tests were done at an NH_3_ adsorption time of 20 min.

### 3.3. Cyclic Test Ability

**Figure 7.**
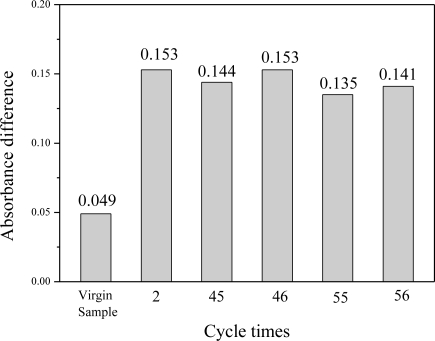
Reversibility tests of the Al-MCM-41(50)/BG material in the absorbance difference for sensing 1.0 ppmv NH_3_ after 20 min of NH_3_ adsorption.

The reversibility of the Al-MCM-41(50)/BG material for ammonia sensing was tested for 71 times by repeated adsorption/desorption of ammonia gas using the same testing material at different ammonia concentrations ranging from 0.25 to 4.3 ppmv. For the same test ammonia concentration of 1.0 ppmv, tests were performed at the beginning (virgin sample), the 2nd, 45th, 46th, 55th and 56th cycle times. Results on the absorbance difference for the same ammonia inlet concentration of 1.0 ppmv are shown in Figure 7.

It is interesting to observe that the absorbance difference of virgin sample is obviously different from the subsequent test results. This phenomenon occurred for all new tested materials. The reason could be due to that a certain percentage of ammonia gas was chemically adsorbed on the sensing material during the first adsorption cycle and forms a very strong chemical bond which could not be desorbed upon heating at 150 °C. After the first adsorption/desorption cycle of the sensing material, the subsequent ammonia adsorption was only due to physical sorption which could be easily desorbed by heating. Thus the subsequent color change rate became fast and stable. One can see that from the 2nd cycle to the 56th cycle, the average value in the absorbance difference was 0.145 ± 0.007 for the five measurement tests, which corresponds to about 5.3% standard deviation. As a result, it is known that the first test result of the virgin sample was not reliable, but after that the material gave stable responses and can be reused for at least tens of cycles.

### 3.4. Calibration for Sub-ppmv Ammonia Gas

**Figure 8.**
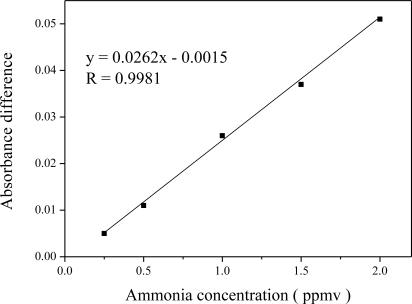
Calibration curve of the Al-MCM-41(50)/BG for sensing sub-ppmv of NH_3_ gas concentration. The test time was 5 min for all NH_3_ concentrations.

In order to understand the sensing ability of Al-MCM-41(50)/BG at different sub-ppmv ammonia concentrations, a calibration curve was established and the result is shown in Figure 8 for ammonia testing concentrations of 0.25 to 2.0 ppmv.

A high linear correlation coefficient (R = 0.9981) was for this test concentration range. By comparing with related research work using silica materials for the same quantification time of 5 min, we can see that this material can detect NH_3_ gas concentrations at least 2 orders lower than those measurable in previous work (145–1,000 ppmv) [23]. In addition to the lower limit of detection, this linear correlation is also better [7,15]. Moreover, the gas quantification time was only 5 min in this study. This means that it has high potential for the industrial application of sensing sub-ppmv ammonia gas concentrations [24]. The method of detection limit (MDL) was calculated by:

(1) MDL=3σ

where σ is the standard deviation of seven repeated measurement data points measured by exposing the sensor to an NH_3_ gas concentration of 0.75 ppmv. The detection limit was found to be 0.185 ppmv. As is known from the slope of regression equation shown in Figure 8, the sensitivity of the detection was 0.0262 for sensing NH_3_ gas in the 0.25∼2.0 ppmv concentration interval.

### 3.5. Effect of the Amount of Material

**Figure 9.**
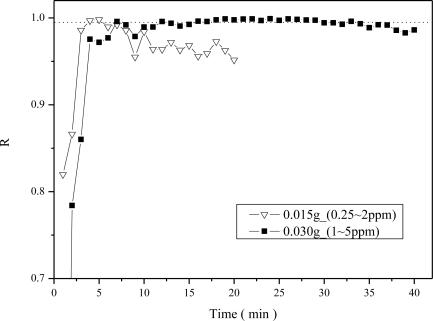
Comparison of the correlation coefficient (R) as a function of NH_3_ sensing time using different amounts of Al-MCM-41(50)/BG materials.

The effect of the amount of material on the linear correlation coefficients (R values) of the calibration curves was evaluated. In the previous results shown in Figure 8, a sample weight of 0.015 g was used. Here the ammonia sensing ability using a thicker sample of 0.030 g in weight was also evaluated and compared with that of the thin sample of 0.015 g in weight. The values of R for the five point calibration curves between 0.25 and 2 ppmv concentrations were obtained initially for the two sample weights of 0.015 and 0.03 g, respectively. However, the thick (0.030 g) sample did not have a clear output signal after a short NH_3_ adsorption time when exposed to 0.25–2 ppmv ammonia concentrations. Hence a higher NH_3_ concentration range of 1 to 5 ppmv was used for the thick (0.030 g) sample. The comparison results in terms of R values at different NH_3_ adsorption times from 2 to 40 min are shown in Figure 9.

The dotted line shown in Figure 9 represents the R value of 0.995, a critical value for indicating a high linear correlation coefficient of the calibration curve. One can see that the required quantitative time with R > 0.995 using the light weight sample (0.015 g) was achieved at a sensing time of only 4 min, which was shorter than that using the heavier weight sample (0.030 g), 8 min. But the time range that R > 0.995 could be maintained was narrower using the 0.015 g light weight sample (4–5 min) as compared to the 0.030 g sample, 12–30 min.

**Figure 10.**
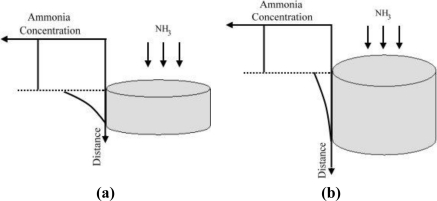
Schematic sketches which show the diffusion effect across the sensing materials of different thickness. **(a)** thin sample (0.015 g). **(b)** thick sample (0.03 g).

The diffusion effect was considered to be responsible for the results observed in Figure 9. As demonstrated in Figure 10, the thicker the material, the longer the diffusion time would be. Besides, as observed from Figure 9, the correlation coefficient decreased after a few minutes for the case of 0.015 g sample, which can be explained by the fact that the thin sample was quickly oversaturated with ammonia molecules. Thus the sensitivity of the thin sample decreased quickly and thus the correlation coefficient was decreased. On the other hand, using the 0.03 g sample which has a thick diffusion layer would result in longer time required for reaching saturated NH_3_ adsorption. Since the buffered capacity using more amount of sensing material was larger, the high correlation coefficient could be maintained for a longer time using the 0.03 g sample.

## 4. Conclusions

An ammonia gas sensing material based on BG dye impregnated on the Al-MCM-41 mesoporous material was proposed. The Al-MCM-41(50)/BG sensing material was orange in color before ammonia gas adsorption, and it changed to blue after exposure to ammonia gas. It was demonstrated by the UV-Vis DRA spectroscopic instrument that the Al-MCM-41(50)/BG is a good sensing material which can detect sub-ppmv ammonia gas concentrations in the range of 0.25 to 2.0 ppmv, with a high linear correlation coefficient. In addition, the response time for quantifying the sub-ppmv ammonia gas concentrations was only a few minutes. The color change process was fully reversible during tens of cyclic tests upon repeated adsorption-desorption of the ammonia gas. The only disadvantage of the material is that without heating, the recovery of the sensor material requires 24 h upon exposure to N_2_ purge gas, which is not practical for instrumentation. So if the material needs to be recovered for cyclic usage, it would require 1 h of heating time at 150 °C which can be engineering designed. The response time and detectable concentration varies with the material weight and thus can be further tuned by adjusting the weight of the sensing material for the detection of different ammonia gas concentrations. Such superiority enables the Al-MCM-41(50)/BG to be an attractive mesoporous material for further development and application in the field monitoring of ammonia gas.
